# Perception about tooth colour and appearance among patients seen in a tertiary hospital, South-West, Nigeria

**DOI:** 10.11604/pamj.2021.38.38.21422

**Published:** 2021-01-14

**Authors:** Deborah Mojirade Ajayi, Shakeerah Olaide Gbadebo, Gbenga Emmanuel Adebayo

**Affiliations:** 1 Department of Restorative Dentistry, College of Medicine, University of Ibadan/University College Hospital Ibadan, Oyo State, Nigeria,; 2 Department of Restorative Dentistry, University College Hospital, Ibadan, Oyo State, Nigeria

**Keywords:** Dental appearance, tooth colour, satisfaction, tooth whitening

## Abstract

**Introduction:**

patients´ satisfaction with their dental appearance and tooth colour is often influenced by certain factors which need to be addressed periodically among different populations.

**Methods:**

a self-administered questionnaire consisting of sociodemographic data, questions on patients´ satisfaction with tooth colour, perceived malalignment of teeth, non-aesthetic anterior tooth-coloured restoration and presence of tooth fracture were distributed. Data collected was analysed using Statistical Package for Social Sciences (IBM, SPSS version 20). Chi square was used to test the statistical differences at a significance of p> 0.05.

**Results:**

a total of 410 patients (M=147, F=263) participated in the study. About 73% had tertiary education while 36.3% were within the modified ISCO-08 Group 2. The respondents that were satisfied with the general dental appearance and tooth shade were 66.3% and 63.5% respectively. More males (65.1%) than females (62.7%) were satisfied with tooth colour while more females (69.1%) were satisfied with dental appearance. The older age group were more satisfied with dental appearance and tooth colour. Awareness of tooth whitening (Over 80%) and the desire to undergo tooth whitening was more among the post-secondary individuals. More of dental patients (73.1%) than medical (59.2%) were satisfied with teeth appearance (p=0.003).

**Conclusion:**

patients are increasingly aware of their dental appearance/tooth colour and the need to improve it with tooth bleaching and/or orthodontic treatment. Female were more dissatisfied with their tooth colour but more satisfied with their dental appearance than the male. Older people were more satisfied with their dental appearance and tooth colour compared to younger age group.

## Introduction

Aesthetics is an important aspect of modern society because it defines one´s personality. Individuals with positive attitude towards their teeth (colour and shape), and smile may show confidence and be extroverts while individuals with discoloured, missing or fractured teeth may on the other hand be withdrawn because of their teeth appearance. Recently in dental treatment, increasing emphasis is being laid on aesthetics [1] with the ultimate objective of creating a beautiful smile. This is to provide teeth of pleasing inherent proportions to one another, and a pleasing tooth arrangement in harmony with the gingiva, lips and face of the patient. Dental appearance being an important determinant in attractiveness of the face plays a key role in human social interactions; and among significant factors affecting the dental appearance are tooth colour, shape, quality of restoration, position and general arrangement of the teeth especially in the anterior region [2]. Natural tooth colour ranges from greyish white to yellowish white, however, a lot of people desire brighter white teeth, which shows that tooth colour is a very important factor determining patients´ satisfaction with dental appearance [2,3]. Also, the outward appearance and colour of teeth, both natural and artificial may be determined by the face and lips. Studies amongst adult population in UK, [2] USA, [4] and China [5] reported varying percentages of people´s dissatisfaction with their colour and appearance which ranged from 28% to 52.6%, however, a study [6] conducted in Nigeria showed that 79.4% of the studied population was satisfied with their dental appearance. Though various groups of patients have different attitudes towards the appearance of their teeth, studies [7,8] have found out that females care more about their dental appearance than males making males to be more satisfied with their teeth appearance than females.

The arrangement of teeth, shape and form, untreated dental caries and non-aesthetic or discoloured anterior teeth restorations as well as missing anterior teeth lead to dissatisfaction with dental appearance. [3,9] Though tooth misalignments are not regarded as serious enough dental problem to necessitate treatment by some people, [10,11] others show high need for rearrangement of their teeth to boost their appearance [12]. Similarly, different treatments being sought to improve dental aesthetic such as bleaching/tooth whitening, aesthetic restoration in anterior teeth have been found to increase patient quality of life and psychological status [13,14]. When a patient´s smile is destroyed by dental diseases which include tooth fracture, discoloration, malalignments, caries etc., the result often is loss of self-esteem and possibly damage to his or her overall physical and mental health. It is thus paramount to assess in any population the view or perception of individuals about their dental appearance in order to improve and/or restore their lost self-esteem and improve their quality of life. The assessments of patients´ satisfaction with their dental appearance, and teeth colour as well as the determination of the factors that influence these variables were the objectives of this study.

## Methods

This was a questionnaire-based cross-sectional study, which made use of the modified version of the questionnaires used by Tin OO *et al*. [1] and Poonam [8]. The self-administered questionnaire consisted of questions on sociodemographic, including gender, age, and level of education, as well as question on patients´ satisfaction with current general dental appearance including satisfaction with tooth colour, perceived malalignment of teeth (crowding, poorly aligned or protruding), presence of caries in anterior teeth, non-aesthetic anterior tooth-coloured restoration and presence of tooth fracture. Study participants included all patients attending 2 outpatient clinics at the University College Hospital Ibadan; the General Out Patient clinic (GOPD) and Dental Centre (a first point of call and sorting out clinic for patients presenting for the first time with non-emergency medical ailments/diseases and the first point of call for all dental cases presenting in the University College Hospital respectively). All completed questionnaires were retrieved from the two clinics. Grossly inadequately filled questionnaires were excluded. A modified socioeconomic class grouping based on International Standard Classification of Occupation 2008 (ISCO-08) [15] was used for occupational stratification. The modified socioeconomic class categorization consisted of 4 groups: group I - chief executives, managers, professionals and high-profile businessmen. Group II - technicians (pharmacy, engineering, and medical) Information Communication Technologists, clerks, secretaries and skilled agricultural workers. Group III - cooks, waiters, all artisans, casual workers and traders. Group IV - unemployed graduates, dependents and housewives.

Ethical approval was obtained from the University of Ibadan/University College Hospital Institutional Review Board (UI/UCH IRB) with approval number: UIUCH/EC0155. The data collected was analysed using Statistical Package for Social Sciences (IBM, SPSS version 20). Descriptive statistics was employed and chi square used to test the statistical differences of some responses at a significance of p= 0.05.

## Results

**Figure 1:**
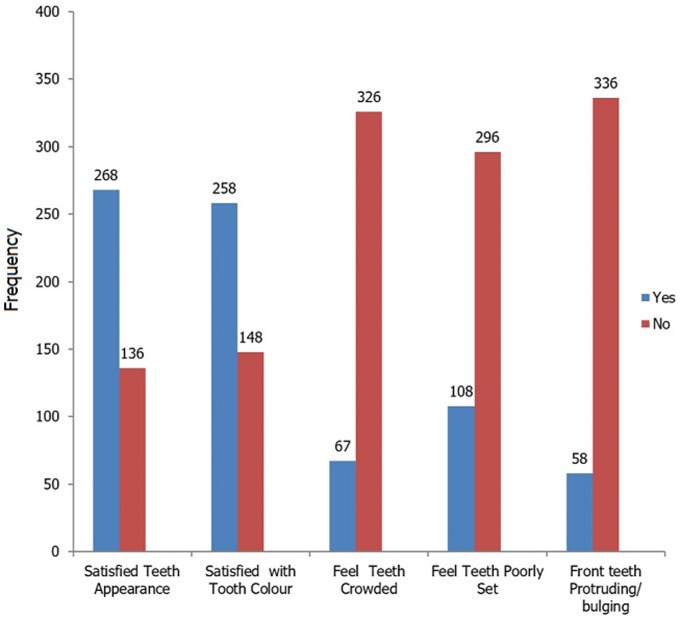
patients' satisfaction with dental appearance and colour

Four hundred and ten patients participated in the study (M=147, F=263) with a mean age of 36.8±14.03 years. Although there were more females than males generally, the M: F among the dental patients was 1: 2.5 compared to 1: 1.3 among the medical patients. The highest proportion (73%) had tertiary education while most of the participants (36.3%) were within the modified ISCO-08 Group 2 which comprised of Technicians, Clerks, Secretaries, Skilled Agricultural workers (Table 1). The findings as shown in Figure 1 revealed that not all the 410 patients in this survey responded to all the questions probing their satisfaction with dental appearance and colour. About 66.3% of those that responded (404) were satisfied with the general dental appearance and almost equal proportion 63.5% of 406 were satisfied with tooth shade. When asked about the teeth arrangement, 26.7%, 17.1% and 14.7% respectively felt that their teeth were crowded, poorly set and had anterior teeth proclination. Relating the gender to the different variables studied as presented in Table 2, more males than females were satisfied with tooth colour (65.1% versus 62.7%), felt teeth were crowded (19.6% versus 15.7%), poorly set (28.3% versus 15.7%), protruding/bulging in the anterior region (16.4% versus 13.8%); had holes (26.7% versus 16.2%, p=0.01); had fractured or missing anterior teeth. However, more females were satisfied with dental appearance (69.1% versus 61.4%) and knew discoloured teeth could be whitened (82.5% versus 79.0). Nevertheless more males had desires to undergo either restorative orthodontic or aesthetic treatment. The wish to have fractured anterior teeth restored was the most common desired among the patients (M=67.4%, F= 68.0 %).

**Table 1:** sociodemographic characteristics of the patients

	Total (N=410)		Dental (N=212)		Medical (N=198)	
	N	%	N	%	N	%
**Gender**						
Male	147	35.9	60	28.3	87	43.9
Female	263	64.1	152	71.7	111	56.1
**Age Group**						
≤ 20	48	11.7	22	10.4	26	13.1
21-40	208	50.7	112	52.8	96	48.5
41-60	121	29.5	69	32.5	52	26.3
≥ 61	21	5.1	5	2.4	16	8.1
No response	12	2.9	4	1.9	8	4.0
**Educational status**						
Primary	32	7.8	22	10.4	10	5.1
Secondary	50	12.2	30	14.2	20	10.1
Post-Secondary	24	5.9	14	6.6	10	5.1
Tertiary	292	73.4	140	66.0	152	76.8
No response	12	2.9	6	2.8	6	3.0
**Occupational Status**						
Group 1 modified ISCO-08	49	12	16	7.5	33	16.7
Group 2 modified ISCO-08	149	36.3	84	39.6	65	32.8
Group 3 modified ISCO-08	80	19.5	54	25.5	26	13.1
Group 4 modified ISCO-08	129	31.5	56	26.4	73	36.9
Missing	3	0.7	2	0.9	1	0.5
**Religion**						
Christianity	323	78.8	153	72.2	170	85.9
Islam	82	20.0	56	26.4	26	13.1
Others	5	1.2	3	1.4	2	1.0
**Tribe**						
Yoruba	314	76.6	159	75.0	155	78.3
Igbo	68	16.6	34	16.0	34	17.2
Hausa	4	1.0	4	1.9	0	0
Nigerian/others	4	1.0	1	0.5	3	1.5
Missing	20	4.9	14	6.6	6	3.0
**Marital Status**						
Single	181	44.1	83	39.2	98	49.5
Married	209	51.0	120	56.6	89	44.9
Separated	4	1.0	1	0.5	3	1.5
Divorced	1	0.2	6	2.8	1	0.5
Widowed	10	2.4	2	0.9	4	2.0
Missing	5	1.2	-		3	1.5

**Table 2:** dental appearance and tooth colour among gender

Variable	Gender							X2	P Value
		Male				Female			
		n	%			n	%		
Satisfied with teeth appearance	YES	89	61.4			179	69.1	2.489	0.115
	NO	56	38.6			80	30.9		
Satisfied with tooth colour	YES	95	65.1			163	62.7	0.228	0.633
	NO	51	34.9			97	37.3		
Felt teeth were crowded	YES	27	19.6			40	15.7	0.953	0.329
	NO	111	80.4			215	84.3		
Felt teeth were poorly set	YES	41	28.3			67	25.9	0.275	0.600
	NO	104	71.7			192	74.1		
Felt front teeth were protruding /bulging	YES	23	16.4			35	13.8	0.505	0.478
	NO	117	83.6			219	86.2		
Felt dental caries/holes in teeth	YES	39		26.7		42	16.2	6.527	0.011
	NO	107		73.3		218	83.8		
Had non aesthetic restoration in the anterior teeth	YES	11		7.60		16	5.8	0.294	0.588
	NO	133		92.4		241	94.2		
Perceived fractured front teeth	YES	32		21.9		38	14.5	3.626	0.057
	NO	114		78.1		224	85.5		
Had missing front teeth	YES	18		12.3		22	8.4	1.607	0.205
	NO	128		87.7		239	91.6		
Wish to restore back fractured teeth	YES	95		67.4		170	68.0	0.016	0.899
	NO	46		32.6		80	32.0		
Do you know discoloured teeth can be whitened	YES	113			79.0	207	82.5	0.710	0.399
	NO	30			21.0	44	17.5		
Wish to undergo orthodontic treatment	YES	60			46.2	83	35.6	3.876	0.049
	NO	70			53.8	150	64.4		
Wish to undergo tooth whitening treatment	YES	89			63.1	142	57.5	1.181	0.277
	NO	52			36.9	105	42.5		
Wish to have dental crown if needed	YES	43			32.6	63	28.1	0.787	0.375
	NO	89			67.4	161	71.9		
Wish to have tooth colour fillings	YES	51			37.5	75	32.1	1.137	0.286
	NO	85			62.5	159	67.9		
Wish to have missing teeth replaced	YES	46			33.8	65	27.8	1.497	0.221
	NO	90			66.2	169	72.2		

It seems the older the age, the greater is the satisfaction with either the dental appearance or tooth colour. The feeling of having protruded anterior teeth was seen more among the youngest age group studied (< 20) compared to other age groups, this was found to be statistically significant (p=0.005). The desire to undergo orthodontic treatment was equally high among the very young and the very old. However, the knowledge of tooth whitening was highest among the youngest age group (Table 3). The association between different educational levels and satisfaction with dental appearance, tooth colour, feeling of presence of crowded teeth as well as presence of missing anterior teeth were all statistically significant (p= 0.013, 0.001, 0.000, 0.031 respectively) with greater tendency to have satisfaction with dental appearance and colour seen among the primary and secondary school holders respectively while feeling of crowded teeth and presence of missing anterior teeth were more in those with post-secondary. The least percentage of missing anterior teeth was seen in people with tertiary education. Over 80% of those with post-secondary/tertiary education knew discoloured teeth could be whitened, however, the desire to undergo orthodontic treatment, tooth whitening and crowning was more among the post-secondary individuals than the others (Table 4). Satisfaction with dental appearance and tooth colour was more prevalent among the occupational group 3, so also was the desire to undergo tooth whitening, dental crowning, tooth colour fillings (p=0.02) and replacement of missing teeth. Comparing dental appearance with tooth colour satisfaction level of dental and medical patients, there were significant differences only with the general dental appearance (p=0.003), the feeling of having poorly set teeth, (0.000), and feeling of having bulging or protruding teeth (p=0.001) between these two groups of patients. More of dental patients (73.1%) than medical (59.2%) were satisfied with teeth appearance whereas more of medical than dental felt they had poorly set teeth (Medical= 35.6%, Dental=18.6%), protruding front teeth (Medical=20.5% Dental =9.3%). Fractured front teeth (Medical=22.3%, Dental 12.3%) (Table 5). The wish to undergo orthodontic treatment crowning of teeth and missing tooth replacement was greater among the medical patients and these associations were statistically significant (p=0.018, 0.009, 0.000 respectively). The perception of fractured front teeth was also significantly more among the medical patients (p=0.005) (Table 6).

**Table 3:** dental appearance, tooth colour and desired aesthetic treatment among age groups

Variable	Age Group											X2	P Value
		≤ 20YRS		21 - 40YRS			41 - 60YRS		≥ 61YRS				
		n	%	n		%	n	%	n		%		
Satisfied with teeth appearance	YES	24	51.1	144		69.9	79	66.4	16		80.0	7.742	0.052
	NO	23	48.9	62		30.1	40	33.6	4		20.0		
Satisfied with tooth colour	YES	27	56.2	135		65.5	76	63.9	15		71.4	1.963	0.580
	NO	21	43.8	71		34.5	43	36.1	6		28.6		
Felt teeth were crowded	YES	12	25.5	32		15.9	16	13.9	6		33.3	6.643	0.84
	NO	35	74.5	169		84.1	99	86.1	12		66.7		
Felt teeth were poorly set	YES	15	31.9	57		27.8	24	20.0	7		35.0	4.245	0.236
	NO	32	68.1	148		72.2	96	80.0	13		65.0		
Felt front teeth were protruding/ bulging	YES	14	29.2	24		12.1	17	14.8	0		00.0	12.755	0.005
	NO	34	70.8	175		87.9	98	85.2	20		100.0		
Felt dental caries/holes in teeth	YES	4	8.30	43		21.0	25	20.8	7		33.3	6.578	0.087
	NO	44	91.7	162		79.0	95	79.2	14		66.7		
Had non aesthetic restoration in the anterior teeth	YES	4	8.30	12		5.90	8	6.90	2		9.5	0.699	0.873
	NO	44	91.7	192		94.1	108	93.1	19		90.5		
Perceived fractured front teeth	YES	7	14.6	33		16.0	22	18.2	7		33.3	4.294	0.231
	NO	41	85.4	173		84.0	99	81.8	14		66.7		
Had missing front teeth	YES	4	8.5	20		9.70	12	10.0	3		15.8	0.570	0.903
	NO	43	91.5	187		90.3	108	90.0	18		84.2		
Wish to restore back fractured teeth	YES	32	68.1	134		67.3	75	65.8	15		78.9	1.297	0.730
	NO	15	31.9	65		32.7	39	32.2	4		21.1		
Do you know discoloured teeth can be whitened	YES	42	91.3	164		81.2	89	76.7	16		84.2	4.712	0.194
	NO	4	8.70	38		18.8	27	23.3	3		15.8		
Wish to undergo orthodontic treatment	YES	19	44.2	74		39.4	36	35.3	8		44.4	1.309	0.727
	NO	24	55.8		114	60.6	66	64.7		10	55.6		
Wish to undergo tooth whitening treatment	YES	27	58.7	119		60.1	65	57.5	11		57.9	0.212	0.976
	NO	19	41.3	79		39.9	48	42.5	8		42.1		
Wish to have dental crown if needed	YES	11	26.2	62		34.6	26	24.8	4		22.2	4.017	0.260
	NO	31	73.8	117		65.4	79	75.2	14		77.8		
Wish to have tooth colour fillings	YES	10	23.3	61		32.4	40	37.0	9		47.4	4.365	0.225
	NO	33	76.7	127		67.6	68	63.0	10		52.6		
Wish to have missing teeth replaced	YES	11	25.6	58		30.7	31	28.7	9		50.0	3.891	0.274
	NO	32	74.4	131		69.3	77	71.3	9		50.0		

**Table 4:** self-reported dental appearance, teeth colour satisfaction and desired aesthetic among different educational level

Variable	Educational level									X2	P Value
		Primary		Secondary		Post-Secondary		Tertiary			
		n	%	n	%	n	%	n	%		
Satisfied with teeth appearance	YES	24	77.4	35	74.5	9	39.1	192	66.0	10.735	0.013*
	NO	7	22.6	12	25.5	14	60.9	99	34.0		
Satisfied with tooth colour	YES	26	74.3	40	80.0	10	41.7	174	60.4	16.331	0.001*
	NO	6	25.5	10	20.0	14	58.3	114	39.6		
Felt teeth were crowded	YES	9	30.0	12	26.1	8	40.0	35	12.2	18.711	0.000*
	NO	21	70.0	34	73.9	12	60.0	252	87.8		
Felt teeth were poorly set	YES	9	28.1	16	32.7	9	39.1	72	25.0	3.113	0.374
	NO	23	71.9	33	67.3	14	60.9	216	75.0		
Felt front teeth were protruding bulging	YES	7	21.9	8	17.8	4	18.2	38	13.4	2.228	0.526
	NO	25	78.1	37	82.2	18	81.8	246	86.6		
Felt dental caries/holes in teeth	YES	6	18.8	5	10.2	6	26.1	61	21.0	3.715	0.2943
	NO	26	81.2	44	89.8	17	73.9	229	79.0		
Had non aesthetic restoration in the teeth	YES	3	9.40	1	2.10	4	17.4	18	6.20	6.345	0.96
	NO	29	90.6	47	97.9	19	82.6	270	93.8		
Perceived fractured front teeth	YES	4	12.5	5	10.0	6	33.3	53	18.3	3.582	0.310
	NO	28	87.5	45	90.0	18	66.7	237	81.7		
Had missing front teeth	YES	5	16.1	6	12.2	6	25.0	23	7.90	8.882	0.031*
	NO	26	83.9	43	87.8	18	75.0	268	92.1		
Wish to restore back fractured teeth	YES	17	58.6	32	68.1	15	62.5	193	68.7	1.501	0.682
	NO	12	41.4	15	31.9	9	37.5	88	31.3		
Do you know discoloured teeth can be whitened	YES	21	70.0	38	79.2	17	85.0	234	82.4	3.043	0.385
	NO	9	30.0	10	20.8	3	15.0	50	17.6		
Wish to undergo Orthodontic treatment	YES	14	48.3	15	40.5	11	52.4	98	36.7	3.258	0.353
	NO	15	51.7	22	59.5	10	47.6	169	63.3		
Wish to undergo treatment tooth whitened	YES	18	60.0	27	56.2	16	72.7	163	58.6	1.885	0.597
	NO	12	40.0	21	43.8	6	27.3	115	41.4		
Wish to have dental crown if needed	YES	5	17.9	13	28.9	9	47.4	75	29.5	4.752	0.191
	NO	23	82.1	32	71.1	10	52.6	179	70.5		
Wish to have tooth colour fillings	YES	14	48.3	16	34.0	10	47.6	82	45.3	5.309	0.151
	NO	15	51.7	31	66.0	11	52.4	181	54.7		
Wish to have missing teeth replaced	YES	13	46.4	17	37.0	5	22.7	73	27.7	5.906	0.116
	NO	15	53.6	29	63.0	17	77.3	191	72.3		

* Statistically significant

**Table 5:** occupational level among dental and medical patients

Variable	Occupation									X2	P Value
		Group 1 Modified ISCO-08		Group 2 Modified ISCO-08		Group 3 Modified ISCO-08		Group 4 Modified ISCO-08			
		n	%	n	%	n	%	n	%		
Satisfied with teeth appearance	YES	29	59.2	99	66.9	56	72.7	81	63.8	2.901	0.407
	NO	20	40.8	49	33.1	21	27.3	46	36.2		
Satisfied with tooth colour	YES	29	60.4	87	59.2	56	70.9	83	64.3	3.260	0.353
	NO	19	39.6	60	40.8	23	29.1	46	35.7		
Felt teeth were crowded	YES	4	8.30	18	12.4	13	18.6	30	23.6	8.895	0.031*
	NO	44	91.7	127	87.6	57	81.4	97	76.4		
Felt teeth were poorly set	YES	11	22.9	33	22.8	20	25.0	42	32.8	4.075	0.253
	NO	37	77.1	112	77.2	60	75.0	86	67.2		
Felt front teeth were protruding bulging	YES	5	10.2	18	12.8	11	15.1	22	17.2	1.845	0.605
	NO	44	89.8	123	87.2	62	84.9	106	82.8		
Felt dental caries/holes in teeth	YES	13	26.5	29	19.1	14	17.7	23	17.8	1.933	0.586
	NO	36	73.5	117	80.1	65	82.3	106	82.2		
Had non aesthetic restoration in the teeth	YES	3	6.10	7	4.90	6	7.9	11	8.5	1.644	0.649
	NO	46	93.9	137	95.1	70	92.1	118	91.5		
Perceived fractured front teeth	YES	11	22.4	27	18.2	11	13.8	21	16.4	1.777	0.620
	NO	38	77.6	121	81.8	69	86.2	107	83.6		
Had missing front teeth	YES	5	10.2	10	6.70	13	16.5	11	8.70	5.830	0.120
	NO	44	89.8	139	93.3	66	83.5	116	91.3		
Wish to restore back fractured teeth	YES	36	75.0	90	63.8	50	69.4	86	67.7	2.224	0.527
	NO	12	25.0	51	36.2	22	30.6	41	32.3		
Do you know discoloured teeth can be whitened	YES	40	81.6	112	78.9	59	77.6	106	85.5	2.617	0.455
	NO	9	18.4	30	21.1	17	22.4	18	14.5		
Wish to undergo Orthodontic treatment	YES	16	38.1	50	63.0	30	45.5	45	38.5	1.397	0.706
	NO	26	61.9	85	37.0	36	54.5	72	61.5		
Wish to undergo tooth whitening treatment	YES	25	53.2	87	61.3	47	64.4	70	56.9	2.025	0.567
	NO	22	46.8	55	38.7	26	35.6	53	43.1		
Wish to have dental crown if needed	YES	15	34.1	37	29.4	23	34.3	30	25.9	1.917	0.590
	NO	29	65.9	89	70.6	44	65.7	86	74.1		
Wish to have tooth colour fillings	YES	16	35.6	43	31.9	34	47.9	30	25.9	9.883	0.020*
	NO	29	64.4	92	68.1	37	52.1	86	74.1		
Wish to have missing teeth replaced	YES	12	28.6	42	30.7	27	38.0	28	23.7	4.465	0.215
	NO	30	71.4	95	69.3	44	62.0	90	76.3		

ISCO-08: International Standard of Classification of Occupations 2008 *Statistically significant

**Table 6:** self-reported satisfaction, presence of dental anomalies and desire to undergo aesthetic treatment among dental and medical patients

Variable		Dental Patients		Medical Patients		X2	P Value
		n	%	n	%		
Satisfied with teeth appearance	YES	152	73.1	116	59.2	8.72	0.003*
	NO	56	26.9	80	40.8		
Satisfied with tooth colour	YES	131	62.4	127	64.8	0.255	0.344
	NO	79	37.6	69	35.2		
Felt teeth were crowded	YES	34	16.7	33	17.5	0.44	0.470
	NO	170	83.3	156	82.5		
Felt teeth were poorly set	YES	39	18.6	69	35.6	1.87	0.00*
	NO	171	81.4	125	64.4		
Felt front teeth were protruding bulging	YES	19	9.3	39	20.5	9.85	0.001*
	NO	185	90.7	151	79.5		
Felt dental caries/holes in teeth	YES	39	18.5	42	21.6	0.592	0.26
	NO	172	81.5	153	78.5		
Had non aesthetic restoration in the teeth	YES	10	5.30	17	8.80	2.47	0.85
	NO	179	94.7	177	91.2		
Perceived fractured front teeth	YES	26	12.3	44	22.3	7.19	0.005*
	NO	185	87.7	153	77.7		
Had missing front teeth	YES	16	7.50	24	12.3	2.49	0.79
	NO	195	92.5	172	87.8		
Wish to restore back fractured teeth	YES	129	63.9	136	72.0	2.93	0.54
	NO	73	36.1	53	28.0		
Do you know discoloured teeth can be whitened	YES	166	80.2	154	82.4	0.300	0.338
	NO	41	19.8	33	17.7		
Wish to undergo Orthodontic treatment	YES	63	33.9	80	45.2	4.874	0.018*
	NO	123	66.1	97	54.8		
Wish to undergo tooth whitening treatment	YES	122	60.1	80	45.2	0.056	0.447
	NO	81	39.9	97	54.8		
Wish to have dental crown if needed	YES	45	24.1	61	36.1	6.145	0.009*
	NO	142	75.9	108	63.9		
Wish to have tooth colour fillings	YES	68	34.9	58	33.1	0.123	0.405
	NO	12	65.1	117	66.9		
Wish to have missing teeth replaced	YES	41	21.1	70	39.8	15.266	0.00
	NO	153	78.9	106	60.2		

* Statistically significant

## Discussion

Dental aesthetics has increasingly become a concern among patients and clinicians. This is because physical appearance plays a key role in social interaction and the smile and teeth are important features in determining facial attractiveness [16]. Evaluating the level of satisfaction with dental appearance and tooth shade by the patients though subjective, may give an idea of how much individuals place on their dental aesthetics. The present study revealed that the level of satisfaction with dental appearance and aesthetics among the participants was 66.3%. This finding is similar to what was recorded by Qualtruogh *et al*. [2] (62.7%) but higher than the observation of Tin-Oo *et al*. [1] (47.2%), Akarslan *et al*. [9] (57.3%) and Strajnic *et al*. [17] (58.1%). However Azodo *et al*. [6] in Nigeria reported 79.4% and Meng *et al*. [18] in Florida and Alkhatib *et al.*[19] in the UK found it to be 76% and 75% respectively. In Nigeria, many people tend to be satisfied with their teeth appearance, possibly due to financial incapability or unaffordability of aesthetic dental treatment. On the other hand, people in developed countries like USA and UK with stable economy could afford orthodontic treatment and other aesthetic procedures early enough to correct any teeth derangement and discolouration, and therefore will more likely be satisfied with their dental appearance at a later age. Furthermore, this disparity in satisfaction in different population may also be possibly related to the fact that the perception of dental appearance may be influenced by cultural factors and can even be changed within the same population over time [20]. The dissatisfaction with tooth shade in this study was found to be 36.5% which is closely related to 34% seen in adults in the USA [4] and 31.6% seen in North America [21]. The main reason for dissatisfaction with dental appearances among participants in some studies [1,3,22] was tooth colour dissatisfaction. This factor in addition to improved awareness of tooth bleaching may have contributed to increasing prevalence of the population seeking tooth whitening. Our finding of higher prevalence of dissatisfaction with tooth colour among females than male is in agreement with previous studies [1,9,17]. Though in contrast, the satisfaction with dental appearance was higher in females than in males. This latter finding is in agreement with previous authors [3] but in disparity with most studies where females were found to be less satisfied with dental appearance [1,7,17]. No significant difference was found in some studies [5,9,20]. However, psychological research on general body satisfaction has found females to be more sensitive regarding their own appearance than males [23].

It could be deduced that the higher dissatisfaction with dental appearance among males could be due to tooth arrangement problems as more males than females, felt that their front teeth were crowded, poorly set or bulging consequently, they were more willing to undergo orthodontic, restorative or any aesthetic treatment. This is not unexpected since malocclusion could also determine dental appearance. Teeth arrangement is a factor correlated to a harmonious smile and attractiveness [24] and divers types of malocclusion could produce dissatisfaction with dental appearance [25]. The findings from this study is in agreement with some previous studies [19,18] which reported that the older patients were more satisfied with their dental appearance or tooth colour. This is probably due to the fact that older people may care less about their appearance, placing priority or emphasis on other issues of life which they might count to be of more importance. Meng *et al*. [18] found that 75% of older respondents were satisfied with appearance and colour. Therefore, age had an impact on dissatisfaction with dental aesthetics, with younger age groups being more dissatisfied, probably as a result of cognitive factors other than social and cultural ones [19]. This may also be caused by media influences, since the young adults are more vulnerable to the effects of the media than older generations [16]. The greater tendency to have satisfaction with both dental appearance and tooth colour seen among the primary and secondary school holders could be due to the low level of exposure. This finding is however not in agreement with the previous studies [5,9] where patients with high level of education were found to be more satisfied with the colour of their teeth, though study by Tin OO *et al*. [1] did not observe any impact of education on satisfaction with tooth colour or dental appearance. As expected, those with tertiary education had the least number of missing anterior teeth. Spaces created by missing anterior teeth could be so unsightly that this should be a major concern to people irrespective of level of education. Nevertheless, individual´s perception of what contributes to facial attractiveness differs. Furthermore, patients with post-secondary education had the greatest desire to undergo orthodontic treatment, tooth whitening and crowning possibly because of the greater perception of crowded teeth, poorly set teeth, carious anterior teeth, fractured and missing anterior teeth. This felt need may motivate them to willingly undergo the dental treatment if they have the economic power.

This study also evaluated the correlation between occupation and the parameters studied which may not have been previously recorded. It was found that 59.2% of the executives and high-profile professionals and business tycoons were satisfied with their teeth appearance as compared to 72.7% in Group 3. One reason for this may be the caliber of people or the company of peoples those in Group I are likely to be relating with. Though the occupational group 4 had many features of unacceptable occlusion the desire to undergo orthodontic treatment was very low (38.5%) among them and this may not be unconnected to their possible low economic status. Findings in this study also show that there were significant difference in the satisfaction of dental and medical patients as more of dental patients than medical patients were satisfied with teeth appearance. More of medical patients felt they had poorly set and protruding front teeth. These observations pointed to the fact that the dental health of the dental patients appeared to be better than that of those patients that were attending the hospital for medical reasons. This is probably because the dental patients are more aware and possibly have started receiving aesthetic dental treatments that have improved their dental appearance to certain extent.

## Conclusion

Within the limitations of this study, it could be concluded that: patients are increasingly aware of their dental appearance/tooth colour and the need to improve it with tooth bleaching and/or orthodontic treatment; female are more dissatisfied with their tooth colour but more satisfied with their dental appearance than the male; older people are more satisfied with their dental appearance and tooth colour compared to the younger age group.

### What is known about this topic

- Studies have shown that males are more satisfied with their tooth colour and appearance whereas females are more conscious and difficult to satisfy;
- Dental treatments that improve anterior teeth aesthetics have been found to improve the quality of life and psychosocial well-being of people.

### What this study adds

- Satisfaction with dental appearance is better in patients that are aware and seek dental treatment than those that are not;
- In this study, medical patient showed a greater desire to have dental treatment that will improve aesthetic such as restoration and orthodontic treatment. Thus, this shows the need for more dental education and information in our environment.
